# Syndecan‐4 and stromal cell‐derived factor‐1 alpha functionalized endovascular scaffold facilitates adhesion, spreading and differentiation of endothelial colony forming cells and functions under flow and shear stress conditions

**DOI:** 10.1002/jbm.b.35170

**Published:** 2022-10-08

**Authors:** Yidi Wu, Saami K. Yazdani, Johanna Elin Marie Bolander, William D. Wagner

**Affiliations:** ^1^ Department of Plastic and Reconstructive Surgery Wake Forest University School of Medicine Winston‐Salem North Carolina USA; ^2^ Biomedical Engineering and Sciences Virginia Tech ‐ Wake Forest University School Winston‐Salem North Carolina USA; ^3^ Department of Engineering Wake Forest University Winston‐Salem North Carolina USA; ^4^ Wake Forest Institute for Regenerative Medicine, Wake Forest School of Medicine Medical Center Boulevard Winston‐Salem North Carolina USA

## Abstract

Acellular vascular scaffolds with capture molecules have shown great promise in recruiting circulating endothelial colony forming cells (ECFCs) to promote in vivo endothelialization. A microenvironment conducive to cell spreading and differentiation following initial cell capture are key to the eventual formation of a functional endothelium. In this study, syndecan‐4 and stromal cell‐derived factor‐1 alpha were used to functionalize an elastomeric biomaterial composed of poly(glycerol sebacate), Silk Fibroin and Type I Collagen, termed PFC, to enhance ECFC‐material interaction. Functionalized PFC (fPFC) showed significantly greater ECFCs capture capability under physiological flow. Individual cell spreading area on fPFC (1474 ± 63 μm^2^) was significantly greater than on PFC (1187 ± 54 μm^2^) as early as 2 h, indicating enhanced cell–material interaction. Moreover, fPFC significantly upregulated the expression of endothelial cell specific markers such as platelet endothelial cell adhesion molecule (24‐fold) and Von Willebrand Factor (11‐fold) compared with tissue culture plastic after 7 days, demonstrating differentiation of ECFCs into endothelial cells. fPFC fabricated as small diameter conduits and tested using a pulsatile blood flow bioreactor were stable and maintained function. The findings suggest that the new surface functionalization strategy proposed here results in an endovascular material with enhanced endothelialization.

## 1 INTRODUCTION

Myocardial infarction attributed to coronary artery disease (CAD) remains the leading cause of death worldwide.^1^ Percutaneous coronary intervention and coronary artery bypass graft (CABG) surgeries are the most common treatments for CAD patients and remains the best practice for patients with complex multivessel disease.^2^ While saphenous vein and internal mammary artery grafts are gold standard for CABG surgery, problems associated with these autologous grafts include invasive harvesting procedure and limited availability. Expanded polytetrafluoroethylene (ePTFE, Teflon®) and polyethylene terephthalate (Dacron®) grafts have been successfully used clinically in large diameter arterial procedures, but have poor performance in small diameter (<6 mm) CABG surgeries due to the risk for compliance mismatch, thrombosis and neointimal hyperplasia.^3^ Consequently, the unmet clinical need for compatible and reliable vascular grafts for CABG surgeries drives the development of novel endovascular biomaterials.

The rapid formation of an intact functional endothelium is critical for the clinical success of any cell‐free graft once implanted, especially for small diameter grafts. Endothelial colony forming cells (ECFCs) are of prime importance in vascular remodeling and repair and are directly involved in the formation a functional endothelium.^4^ Recent efforts and strategies have been directed toward functionalizing biomaterial surfaces with capture molecules to enhance ECFCs binding capacity since ECFCs circulate at small numbers in the blood.^5^ Antibody‐based strategies (including antibodies against CD34, CD31, vascular endothelial growth factor receptor 2 [VEGFR2] or vWF) have been shown to facilitate the binding of ECFCs to scaffold materials.^6^, ^7^ Although this approach has been used clinically to accelerate endothelization,^8^, ^9^ controversy regarding safety and efficacy remains. Intimal hyperplasia has been observed at the venous anastomosis of anti‐CD34–coated ePTFE grafts after 4 weeks in a porcine arteriovenous model.^10^ In another study, increased density of anti‐VEGFR2 antibody functionalized surfaces resulted in an inhibition of cell proliferation.^11^ Similarly, although ECFC‐specific peptides, aptamers or oligosaccharides have been used as capture molecules, they are not active in promoting cell proliferation and differentiation.

Alternatively, growth factors can be immobilized onto the biomaterial in a physiological manner to recruit ECFCs. It is well known that growth factors are involved in the natural process to mobilize ECFC during vascular injury and promote in situ vascular tissue repair and regeneration.^12^ In addition, the immobilization of growth factors on biomaterials has been reported to facilitate the binding of ECFCs as well as promote cell proliferation and differentiation.^13^ One growth factor Stromal cell‐derived factor‐1 alpha (SDF‐1α) plays a major role in the homing of circulating progenitor cells through the interaction with CXC chemokine receptor 4 (CXCR4). SDF‐1α has been reported to regulate integrin mediated ECFC adhesion to extracellular matrix and differentiation into endothelial cells.^14^ Several groups have reported that the functionalization of biomaterial scaffolds with SDF‐1α successfully recruited ECFCs and promoted vascular tissue repair.^15^, ^16^, ^17^ Clinically available vascular grafts (Gelsoft™ and Polymaille®C) functionalized with SDF‐1α have been shown in ovine models to attract ECFCs, improve endothelialization and reduce intimal hyperplasia and thrombosis.^18^ However, the capture rate of ECFCs was maximum at early phase (after 24 h), and less than 50% of the scaffold was covered with endothelium after 3 months. This was possibly due to the adsorbed SDF‐1α being nonresilient to pulsatile vascular flow or subjected to enzymatic degradation. Another explanation could be that multiple signaling molecules are involved in promoting cell proliferation and differentiation. Local delivery of a single growth factor may appear to be beneficial initially but may not provide the optimum solution in the long term. To achieve sufficient endothelization on scaffold material, a multifunctional and sustainable approach for functionalizing scaffold material may be beneficial.

**FIGURE 1:**
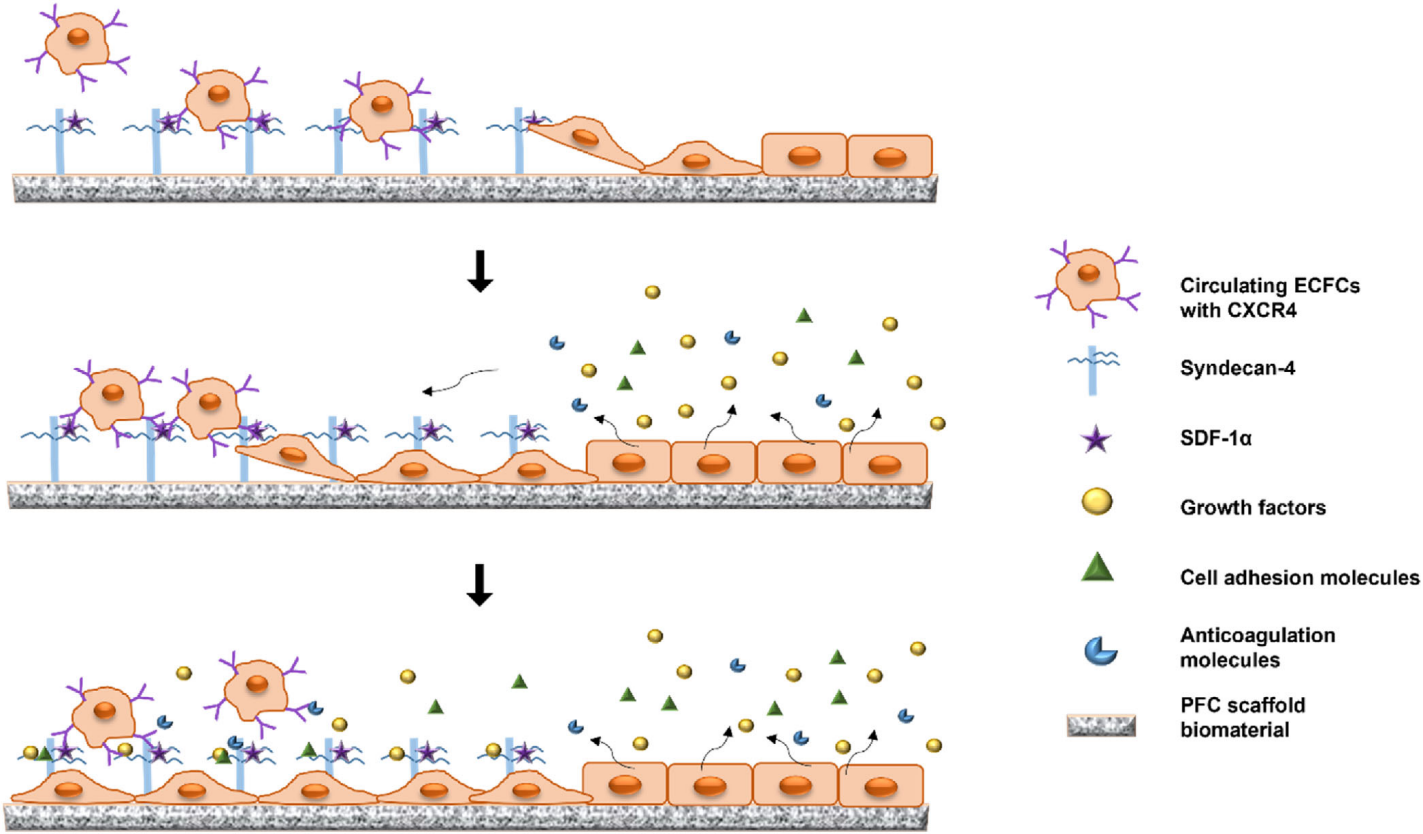
Schematic illustrating the potential initial interaction of syndecan‐4/SDF‐1α with circulating ECFCs (Top) and later syndecan‐4 interaction with molecules (paracrine and/or autocrine) over time. The figure illustrates early events in endothelialization. Not drawn to scale, only a few syndecan‐4 molecules are illustrated.

In this study, syndecan‐4 having multiple ligand binding sites was covalently linked on a biomaterial in order to deliver and attract growth factors to promote endogenous regeneration. This approach is different from existing strategies using either direct adsorption or chemically linked growth factors and may create a unique solution and meet the need for in situ supply of multiple signaling molecules. The molecular diversity of the oligosaccharide sequences on syndecan‐4 makes it a unique candidate to functionalize a variety of scaffold materials and deliver tissue‐specific biochemical cues to repair and regenerate different types of tissues.^19^ For instance, the binding sites that are specific for SDF‐1α, bone morphogen proteins, fibroblast growth factors and vascular endothelial growth factors (VEGF) have been identified in syndecan‐4.^20^, ^21^ We hypothesized that the combination of syndecan‐4 and SDF‐1α may work as a functional layer on biomaterial scaffolds to facilitate the binding of ECFCs (see Figure 1). Moreover, syndecan‐4 has binding sites to retain and present cell‐secreted growth and signaling molecules that are essential for cell proliferation and differentiation. Thus, the syndecan‐4 and SDF‐1α functionalization of the biomaterial may cause enhanced cell spreading and accelerate cell proliferation and differentiation.

In a previous study, an endovascular biomaterial composite of poly(glycerol sebacate), Silk Fibroin and Type I Collagen (PFC) was created. The composition and nanoscale structure of PFC is similar to extracellular matrix of native vascular tissue and has been shown to support the growth of mature endothelial cells eventually forming a confluent monolayer.^22^ PFC is elastic and mechanically durable with a slow degradation rate, which permits vascular regeneration and remodeling in vivo without weakening mechanical properties.^22^ In the presented study, PFC was used as a model scaffold material to determine if functionalization with syndecan‐4 and SDF‐1α would enhance the interaction with ECFCs. It was hypothesized that the functionalization would promote rapid endothelialization by creating a local microenvironment conducive for ECFC adhesion, growth, and differentiation.

## 2 MATERIALS AND METHODS

Poly(glycerol‐sebacate) (PGS) prepolymer was synthesized with sebacic acid purchased from Sigma‐Aldrich (St. Louis, MO) and glycerol purchased from Fisher Scientific (Waltham, MA) using a published protocol.^23^ Type I collagen was purchased commercially from Elastin Products Company, Inc (Owensville, MO). Silk fibroin was extracted from raw silk purchased from Haian Silk Company (Nantong, China) according to published methods.^24^ Human bone marrow derived CD34+ cells were purchased from ATCC (Manassas, VA). 1,1,1,3,3,3‐hexafluoro‐2‐propanol (HFIP) and fibronectin were purchased from Sigma‐Aldrich (St. Louis, MO). Recombinant human SDF‐1α, human sydecan‐4 and Quanti SDF‐1α ELISA kit were obtained from R&D Systems (Minneapolis, MN). EGM™‐2 Endothelial Cell Growth Medium‐2 BulletKit™ was purchased from Lonza (Walkersville, MD). Quant‐iT™ PicoGreen™ dsDNA Assay Kit, 4′,6‐diamidino‐2‐phenylindole (DAPI), Rhodamine Phalloidin and SYTOX™ Green Nucleic Acid Stain were purchased from Invitrogen (Waltham, MA).

### 2.1 Scaffold fabrication and functionalization

PFC was composed of silk fibroin, type I collagen and PGS at a mass ratio of 4.5:4.5:1. All components were dissolved in HFIP at 10% w/v ratio for electrospinning as published previously.^22^ PFC polymer solution was stirred overnight and then loaded into a 5 ml syringe with 18‐gauge blunt tip needle. Then a 25 kV voltage was applied to the needle tip and the solution was electrospun onto a collector to obtain random fibers at 2 ml/h. For cell morphology, spreading and differentiation studies, PFC solutions were electrospun as thin sparce fibers onto 15 mm diameter round glass cover slips for 10 min. These preparations have significant areas where fibers do not cross or interact with other fibers and are useful to observe the interaction of individual cells with a length of an individual fiber. For cell capture and cell coverage studies, PFC was prepared as a thicker mat of fibers by electrospinning onto an aluminum foil collector for 60 min. Fiber mats were physically removed from the collector for use. For pulsatile flow bioreactor study, PFC was fabricated as vascular conduits by electrospinning onto a 4‐mm diameter metal rod rotating at 120 rpm for 60 min. After electrospinning, the material was removed from the collector and incubated in the oven at 120°C for 48 h to polymerize PGS. Then the material was treated with 1.5% glutaraldehyde vapor overnight to crosslink the proteins. PFC was then functionalized with syndecan‐4 and SDF‐1α and termed fPFC.^25^ Briefly, PFC was saturated with 0.8 μg syndecan‐4/cm^2^ PFC using the two‐step NHS/EDC method and rinsed with Na_2_HPO_4_ and PBS.^25^ The scaffolds were washed three times with PBS and saturated with 0.8 μg SDF‐1α/cm^2^ PFC for 2 h at 37°C with agitation. The presence of syndecan‐4 and SDF‐1α on PFC was confirmed using ELISA.

### 2.2 Cell culture and characterization

Human bone marrow derived CD34+ cells were cultured in fibronectin‐coated (1 μg/cm^2^) tissue culture flasks at 37°C in EBM‐2 with endothelial growth medium‐2 SingleQuots™ Supplements. Medium was changed after 3 days. Cell colonies appeared after 7 days of culture. To confirm identification of ECFCs the cells were characterized by immunostaining of CXCR4 (CXCR4, receptor specific for SDF‐1α) and CD34 (marker of vascular endothelial progenitor cells) (Images not shown). ECFCs were plated at 5000 cells/cm^2^ and incubated for 24 h. Then cells were fixed with 4% paraformaldehyde (PFA) for 15 min and blocked with 1% BSA overnight at 4°C. Cells were incubated with rabbit anti‐CXCR4 primary antibody from Thermo Scientific (1:200, Waltham, MA) or with anti‐CD34 primary antibody Abcam (1:100, Cambridge, MA) for 1 h at 37°C, and then incubated with AlexaFluor 488 goat antirabbit IgG secondary antibody from Abcam (1:1000, Cambridge, MA) for 2 h at 37°C. Cell nuclei were stained with DAPI.

### 2.3 ECFC spreading and morphology

Cell spreading of ECFCs on fPFC was compared with PFC and tissue culture plastic (TCP) (*N* = 3). PFC and fPFC electrospun fiber covered glass coverslips (15 mm in diameter) were fitted into a 24‐well ultra‐low attachment plate. 50,000 ECFCs were added to the plates and cultured in EBM‐2 medium supplemented with 2% fetal bovine serum (FBS). After incubation at 37°C for 24 h or 4 days, cells were fixed with 4% PFA, and stained with Rhodamine Phalloidin and SYTOX green. The morphology of ECFCs on the materials was evaluated using a Keyence fluorescence microscope (Itasca, IL). Cell spreading area was quantified using the BZ‐X800 Viewer software (version 1.1.2.4). Cell spreading at 2 h was quantified using multiple phase contrast images taken from different fields (*N* = 9). Violin plots were generated to determine the frequency of cell sizes within a population of cells. For cells cultured for 4 days, the averaged cell spreading area (ACSA) was quantified using multiple fluorescence images (*N* = 9) and calculated using the following equation: $\text{ACSA}=\frac{\text{Total cell spreading area}}{\text{Total number of cells}}$.

### 2.4 Cell coverage study

To compare cell coverage rates, 10,000 ECFCs were seeded onto PFC or fPFC electrospun mats (6 mm in diameter) and cultured for 7 days at 37°C (*N* = 3). Each sample was then removed from the wells, washed with PBS, and fixed with 2.5% glutaraldehyde. Then samples were dehydrated using a series of ethanol solution (50%, 60%, 75%, 80%, 90%, 95%, 100%) and further dried using hexamethyldisilazane from Thermo Scientific (Waltham, MA), followed by coating with a thin layer of gold. Cell morphology and coverage (*N* = 3) on each sample were evaluated using scanning electron microscopy with the same imaging parameters (Zeiss GeminiSEM 300) and was analyzed with ImageJ.

### 2.5 ECFC capture study

The capture of ECFCs by scaffolds was evaluated under static and flow conditions. For static conditions, the dynamic binding method was used.^17^ PFC and fPFC electrospun mats (6 mm in diameter) were cut and fitted to a 96‐well ultra‐low attachment plate. 35,000 cells/cm^2^ ECFCs were added to the materials and incubated for 4 h at 37°C (*N* = 3). The plate was placed on an orbital shaker at 4 rpm to prevent cell aggregation and to allow cells to dynamically interact with the materials. The surface was rinsed to remove unbound cells. The number of cells bound to materials was quantified using PicoGreen™ DNA quantification. The binding of ECFCs was evaluated under two levels of shear stress using a custom‐built parallel flow chamber to mimic physiological conditions, ranging from 1–6 dynes/cm^2^ (in veins) to 10–20 dynes/cm^2^ (in arteries).^26^, ^27^, ^28^ The wall shear stress (WSS) in the flow chamber was calculated based on the following equation:

$$\tau_{w}=\frac{6\mathrm{\mu Q}}{a^{2}\;b},$$

𝜏_𝑤_, wall shear stress; 𝜇, viscosity of the medium; *Q*, flow rate; *a*, channel height; *b*, channel width.

ECFCs were seeded at a density of 100,000 cells/cm^2^ under either 1 or 10 dynes/cm^2^ and interacted with fPFC for 4 h in the flow system.^29^ The surface was rinsed to remove unbound cells. The number of ECFCs bound to PFC and fPFC was quantified and normalized using PicoGreen™ DNA quantification.

### 2.6 Binding specificity of SDF‐1α to CXCR4

The binding specificity of ECFCs to fPFC was assessed using the CXCR4 antagonist AMD3100 to compete with SDF‐1α (*N* = 3). For this, ECFCs were incubated with AMD3100 (10 μg/ml) for 30 min to block SDF‐1α binding to the CXCR4 receptors.^30^, ^31^ Then AMD3100 treated ECFCs and untreated ECFCs were incubated with PFC or fPFC electrospun mats for 4 h at 37°C on an orbital shaker rotating at 4 rpm. The surface was rinsed to remove unbound cells. The number of ECFCs bounded to the material was quantified using PicoGreen™ DNA quantification.

### 2.7 Selective binding of ECFCs by fPFC

To study SDF‐1α binding specificity for ECFCs, a mixed population of peripheral blood leukocytes was used to evaluate the binding of ECFCs to fPFC. Leukocytes were isolated from fresh pig blood using a Ficoll gradient following manufacturer protocols. ECFCs were labeled with Vybrant™ DiO Cell‐Labeling Solution from Invitrogen (Waltham, MA) according to the manufacturer's protocol. Then DiO labeled ECFCs were mixed with an increasing number of leukocytes (at a ratio of 1:1, 1:10, and 1:100) (*N* = 3) and incubated at 37°C on an orbital shaker rotated at 4 rpm for 4 h. The surface was rinsed to remove unbound cells. The number of ECFCs bound to fPFC was observed using fluorescence microscopy and quantified.

### 2.8 Real‐time PCR analysis

To investigate transcriptional regulation in cells on fPFC, electrospun PFC, and fPFC fiber covered glass coverslips (15 mm in diameter) were fitted into a 24‐well ultra‐low attachment plate (*N* = 4). Next, 50,000 ECFCs were seeded onto the fibers and cultured in EBM‐2 medium supplemented with 2% FBS. Briefly, cells were lysed using TRIzol™ Reagent from Invitrogen (Waltham, MA) at day 4 and 7 of cell culture. mRNA was isolated and prepared using Luna® Universal One‐Step RT‐qPCR Kit from New England Biolabs, Inc (Ipswich, MA) according to the manufacturer's instructions. mRNA transcript analysis for progenitor cell marker *CD34* and the specific endothelial cell marker *platelet endothelial cell adhesion molecule (PECAM‐1/CD31)* and *von Willebrand factor (vWF)* by human ECFCs were determined by real‐time reverse transcription–polymerase chain reaction (RT‐PCR). TaqMan® gene expression assays for human glyceraldehyde‐3‐phosphate dehydrogenase (GAPDH) (Hs02758991_g1), human CD34 (Hs00990732_m1), human CD31 (Hs00169777_m1), human vWF (Hs01109454_m1) were obtained from Thermo Fisher Scientific (Waltham, MA). RT‐PCR was performed with a CFX Connect Real‐Time PCR System from Bio‐Rad Laboratories (Hercules, CA). Results were analyzed by the 2^−ΔΔCt^ method using housekeeping gene GAPDH. Results for cells on fPFC and PFC were normalized to gene expression levels on TCP.

### 2.9 fPFC conduit under physiological arterial flow condition

**FIGURE 2:**
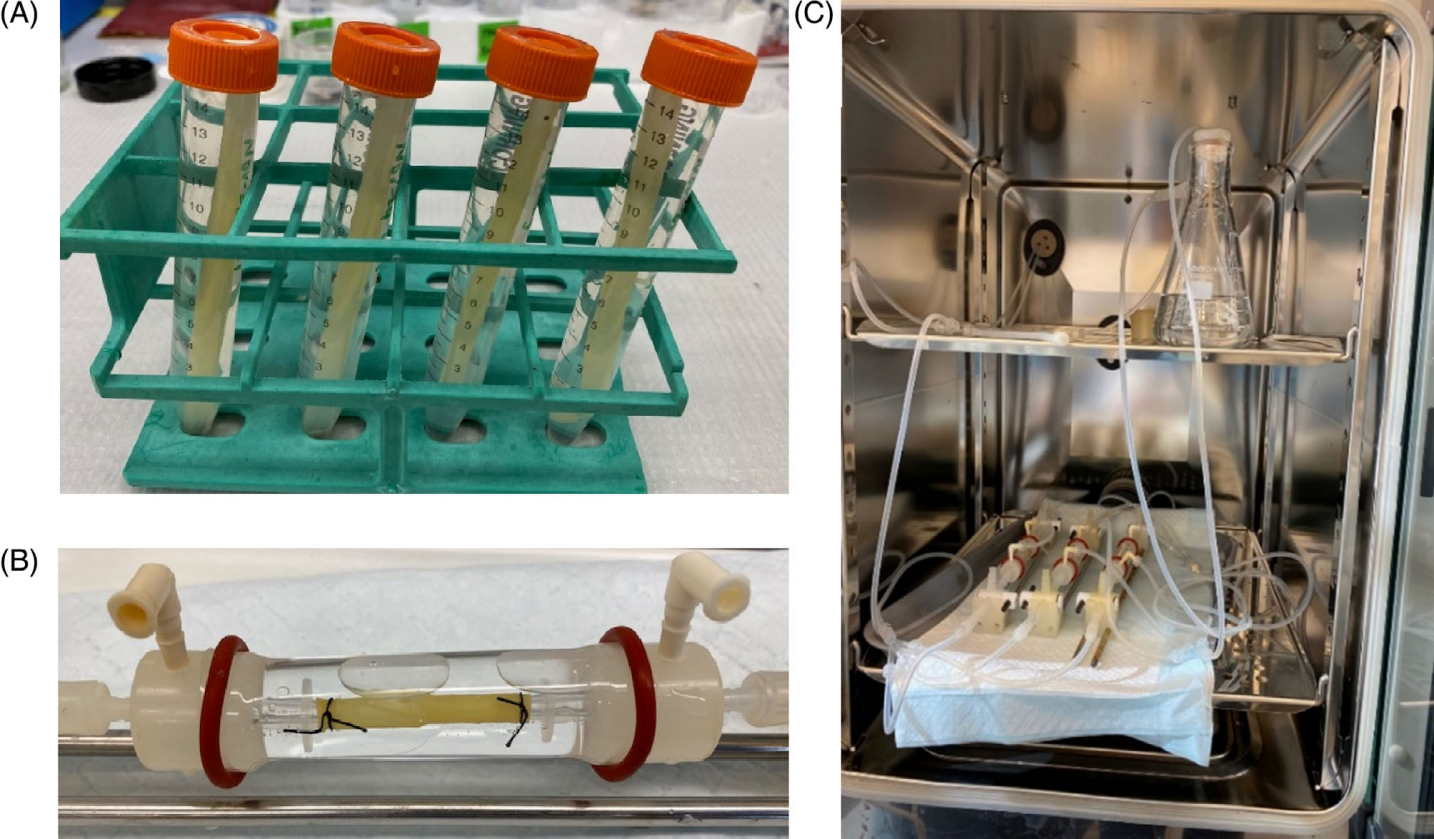
(A) Photograph of electrospun crosslinked fPFC vascular conduits. Homogeneous sections were cut from the center part of these conduits to evaluate in the flow bioreactor. (B) Enlarged view to show vascular conduit secured to flow system. (C) View of three fPFC vascular conduits in parallel in a pulsatile flow bioreactor.

For these studies fPFC was fabricated as vascular conduits with approximately 4 mm inner diameter (ID; Figure 2A). The conduits were hydrated with PBS for 30 min before study and cut into sections of 3 cm in length and inserted into a cannula at both ends (*N* = 3). The ends of the conduit were secured to the cannulas using black silk sutures (Ethicon) to ensure tight connections (see Figure 2B). The ex vivo bioreactor system was kept in an incubator at 37°C and consisted of a flow reservoir (top shelf as shown in Figure 2C), vessel housing compartment (bottom shelf as shown in Figure 2C), gear pump from Ismatec Cole Parmer (Vernon Hills, IL), and a distal flow constrictor. The flow medium was saline with 1% antibiotic‐antimycotic from Gibco (Waltham, Massachusetts). A custom LabVIEW program was used to generate the flow waveform and to control and monitor the flow and pressure within the bioreactor system.^32^ The ID of fPFC vascular conduits was measured using a Chison ECO5 Portable Ultrasound immediately after the flow was started and at 1, 3, 6, and 24 h. After the flow cycle was complete, the vascular conduits were retrieved. The dynamic radial compliance (*C*) was calculated from the ultrasound ID measurements using the following equation:

$$C=\frac{D_{s}-D_{d}}{D_{d}}\times \frac{1}{∆P}\times 10^{4}\;\left(\%/100\;\text{mmHg}\right),$$

$D_{s}$, ID corresponding to systolic pressure; $D_{d}$, ID corresponding to diastolic pressure, ∆P, difference between systolic pressure and diastolic pressure.

Tensile properties of fPFC vascular conduits were determined after being conditioned under physiological arterial flow condition using Instron Mechanical Tester (Instron Corporation) with a 100 N load cell. Ring specimens (5 mm tubular cross‐sections) were placed over the two pins and the material was extended at a rate of 5 mm/min. Stress–strain curves were generated by BlueHill software program (Version 4.25, Instron Corporation). Elastic modulus was calculated by the slope of the elastic region of the curve. The amount of SDF‐1α remaining on the material after arterial flow studies was quantified with ELISA using mouse IgG anti‐SDF‐1α from R&D Systems (1:100, Minneapolis, MN) as the primary antibody and HRP antimouse IgG from Abcam (1:10,000, Cambridge, MA) as the secondary antibody. Conduits incubated at 37°C under static conditions were used as controls.

### 2.10 Statistical analysis

Group means adopted from preexperiment or estimated from published studies and a power of 0.8 were used to calculate sample sizes. Statistical analyses were performed using Prism software (version 9.2.0) with either a Student's *t*‐test or a one‐way analysis of variance depending on study design. Results were presented as mean ± standard error of the mean for each experiment group. If the results were significant (*p* < .05), a Tukey's post hoc test was conducted to separate differences in means.

## 3 RESULTS

**FIGURE 3:**
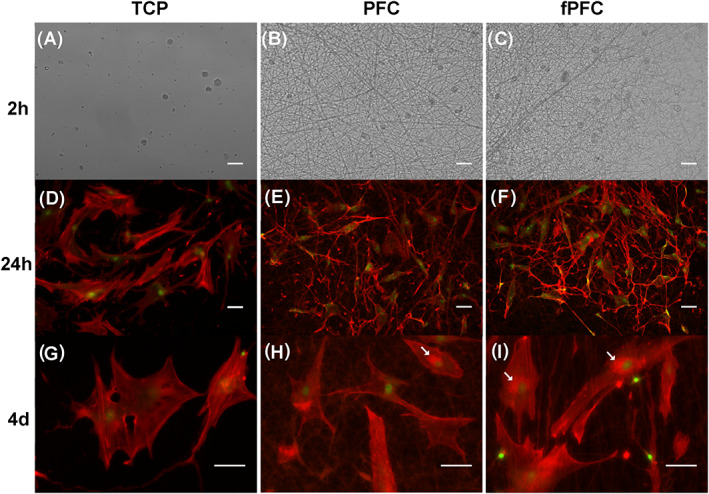
Representative images of ECFC morphology and spreading on TCP, PFC, and fPFC electrospun nanofiber coated coverslips. ECFCs were imaged at 2 h (h) of culture using phase contrast microscopy (A,B,C). At 24 h (D,E,F) and 4 days (d) (G,H,I) of culture, ECFCs were stained and imaged using fluorescence microscopy. Red: Rhodamine‐Phalloidin staining for cytoskeleton, green: SYTOX green for cell nuclei. Scale bar = 100 μm.

Initial studies were designed to evaluate the interaction of ECFC with electrospun fibers to determine if the addition of syndecan‐4 and SDF‐1α to PFC influenced cell attachment and spreading. For these studies, ECFCs were cultured on a sparse layer of fibers to easily monitor the morphology. As shown in Figure 3A–C, ECFCs at 2 h were associated with both PFC and fPFC nanofibers. Cells were spherical in appearance and morphologically similar to cells cultured on TCP.

After 24 h, it was apparent that cells were extended in random directions with both types of electrospun fibers. ECFC cultured on fibers appeared more extended and aligned in the direction of the nanofibers when compared with cells on TCP based on visual inspection (Figure 3D–F). Stress fibers originating from the nuclei in the cells on both types of PFC appeared to be thicker than the fibers in cells grown on TCP, where more sparce and diffuse actin fibers were apparent. The cell–material and cell–cell interaction on both types of PFC appeared similar. At 4 days as shown in Figure 3G–I, the activity of cells cultured on both PFC and fPFC compared with those cultured on TCP appeared to be enhanced as assessed by condensation of actin filament in the pericellular region (see white arrows in Figure 3H,I). Condensation of actin filament in the nuclear region of cells has been reported to be associated with cell differentiation as well as mechanical transduction.^33^

**FIGURE 4:**
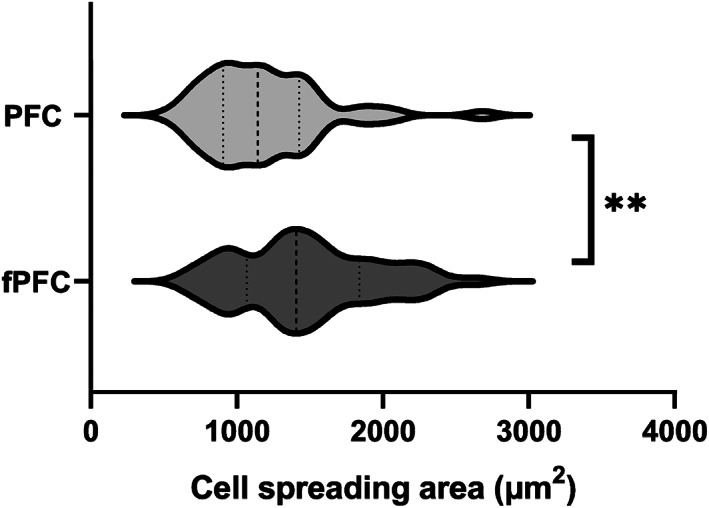
Violin plot of the distribution of ECFC spreading area on PFC and fPFC electrospun nanofiber coated coverslips quantified with multiple phase contrast images at 2 h. The dashed center line represents the median and the dotted lines represent the interquartile ranges. **p < .01.

To quantitatively evaluate the influence of fPFC in the initial interaction and spreading of ECFCs, measurements of cell areas were completed using additional randomly selected sets of images like those shown by Figure 3. Violin plots were used to provide the density distribution of ECFC area after 2 h of cell–material interaction (Figure 4). The wider regions of the plot indicate a higher probability, and the thinner regions represented a lower probability for cell areas.

The median and third quartile of cell spreading areas were greater for fPFC compared with PFC, which suggested a higher population of ECFCs were distributed at the regions of greater spreading areas. The average cell spreading area of ECFCs cultured on fPFC was 24% greater (*p* < .01) than for cells on PFC. fPFC had an average cell spreading area of 1474 ± 63 μm^2^ and PFC was 1187 ± 54 μm^2^ (mean ± SEM).

**FIGURE 5:**
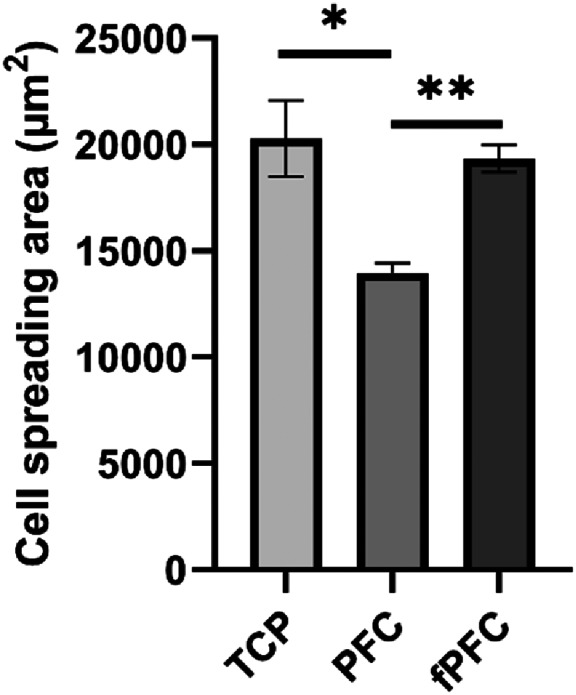
Comparison of average cell spreading area on TCP, PFC, and fPFC electrospun nanofiber coated coverslips quantified with fluorescence images at 4 days (mean ± SEM). These data are calculated from total cell coverage area divided by total cell numbers. *p < .05, **p < .01.

To further evaluate cell spreading beyond 2 h, the cell morphology and spreading were quantified from multiple sets of fluorescent images after 4 days of culture. There was a significant difference (*p* < .01) between the average cell spreading areas for cells grown on PFC and fPFC nanofiber coated coverslips, the average cell spreading area on fPFC was 39% greater than PFC after 4 days of culture (Figure 5). This difference in cell spreading was significantly greater for cells at 4 days compared with 2 h of incubation. These results indicate fPFC positively influenced ECFCs behavior over time.

**FIGURE 6:**
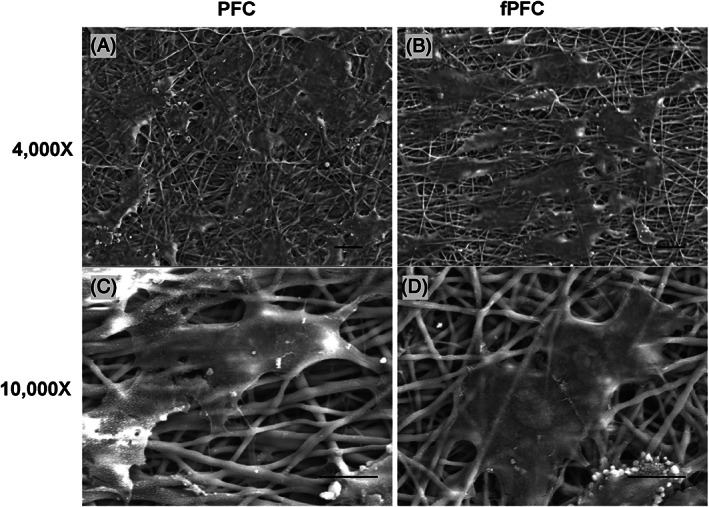
SEM of ECFC‐scaffold interaction at day 7 on PFC (A, C) and fPFC (B, D) electrospun mats at a magnification of 4000× (top, scale bar = 20 μm) and 10,000× (bottom, scale bar = 10 μm).

The ultrastructure of cell–material interaction was evaluated by SEM to evaluate ECFCs interaction and spreading on thick mats of materials. The mats had the same structural properties as PFC described in previous reports from our laboratory.^22^, ^25^ After ECFCs were cultured for 7 days on PFC and fPFC electrospun mats, ECFCs were tightly associated with the nanofibers and covered the surface of PFC as well as fPFC electrospun mats (Figure 6). Similarly, the ultrastructure of cell spreading on fPFC mats appeared greater compared with PFC. The total fraction of area covered by the cells was 47% ± 1.9% for PFC and 59% ± 2.5% for fPFC (*p* < .01). These observations agreed with previous observations on nanofiber coated coverslips demonstrating that cell surface area coverage was greater for cells cultured on fPFC (see Figure 3). Collectively, the findings suggest that over time ECFCs can form an intact cell layer on PFC vascular conduits and coverage is improved by functionalizing with syndecan‐4 and SDF‐1α.

**FIGURE 7:**
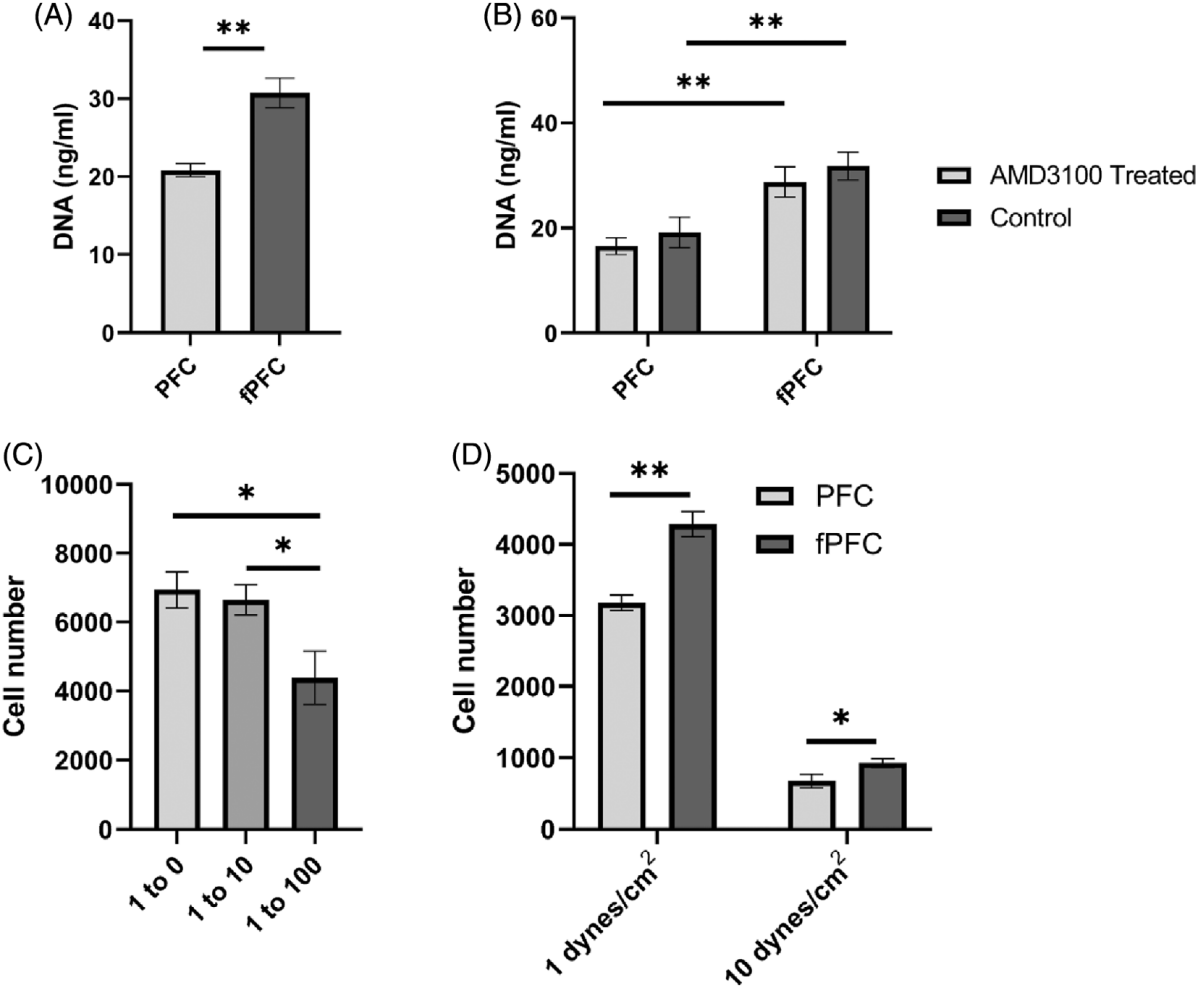
Quantitation of ECFCs captured on PFC and fPFC electrospun scaffolds at 4 h. (A) Binding potential of PFC and fPFC for ECFCs under dynamic binding conditions (on an orbital shaker). (B) Binding specificity of PFC and fPFC to ECFCs in the presence of CXCR4 antagonist AMD3100. (C) ECFC‐fPFC interaction in the presence of competing leukocytes. (D) Quantitation of ECFCs captured on PFC and fPFC under physiological flow conditions. *p < .05, **p < .01.

Further studies were completed to quantitatively evaluate binding of ECFCs to fPFC. In an initial experiment, the extent of binding of ECFCs to PFC and fPFC was evaluated under dynamic binding conditions on an orbital shaker to avoid cell aggregation. fPFC bound about 50% more ECFCs compared with PFC (Figure 7A). The DNA quantification results demonstrate that functionalization significantly facilitate the binding of ECFCs to the biomaterial. To determine that this increase in binding was due to the interaction of CXCR4 on ECFCs and SDF‐1α on fPFC, AMD3100, a potent CXCR4 antagonist and competitor of SDF‐1α was used. After AMD3100 treatment, a nonsignificant reduction on cell binding was observed as determined by the DNA quantification (Figure 7B). A higher concentration of AMD3100 also was evaluated to preclude the possibility of insufficient saturation of CXCR4 receptors on ECFCs, but no difference was observed (data not shown).

Leukocytes have CXCR4 receptors and are present in blood in larger numbers than ECFCs. The binding potential of ECFCs to fPFC in the presence of competing blood leukocytes was evaluated. Binding selectivity for ECFC was evaluated by mixing fluorescently labeled ECFCs with increased levels of added leukocytes (Figure 7C). As determined by fluoresce cell number quantification, 10 times excess leukocytes had no effect on ECFC binding and at 100 times excess reduced binding by only 23%. It is expected this represents a maximum competition since the effect of flow may reduce the binding of potential competing leukocytes due to reduced CXCR4 receptor numbers and binding affinity of those cells.^34^

The study of ECFC adhesion under flow condition provides quantitative information for evaluating and predicting the performance of the material after surgical implantation since shear stress forces will occur at suture sites and at any branch points. To simulate fluid flow shear stress affecting the interaction of cells, the binding of ECFCs to PFC and fPFC was evaluated under low and high shear stress. Based on DNA quantification, more ECFCs were associated with fPFC than PFC at both 1 and 10 dynes/cm^2^ condition (Figure 7D). fPFC captured 37% more ECFCs at 1 dynes/cm^2^ and 32% more ECFCs at 10 dynes/cm^2^ compared with PFC. The results indicate fPFC facilitates the binding of ECFCs to the scaffold material under flow and shear conditions.

To determine if the addition of syndecan‐4 and SDF‐1α enhanced the differentiation of ECFCs, several marker genes were examined using RT‐PCR. These included CD34, vWF, and CD31. The gene expression levels in differentiating cells for these marker genes have been positively related to protein levels and can be used to evaluate cell differentiation.^29^, ^35^ CD34, a hematopoietic progenitor cell marker, is gradually downregulated as progenitor cells differentiate. vWF, a glycoprotein stored in Weibel–Palade bodies in endothelial cells, plays an essential role in hemostasis through the interaction with platelets and clotting factors.^36^ The level of vWF gradually increases as ECFCs proliferate and differentiate into mature endothelial cells. CD 31, a cell surface adhesion molecule involved in endothelial cell adhesion and transendothelial migration, is found at intercellular junctions and plays a significant role in adhesion of endothelial cells and cell–cell interaction.

**FIGURE 8:**
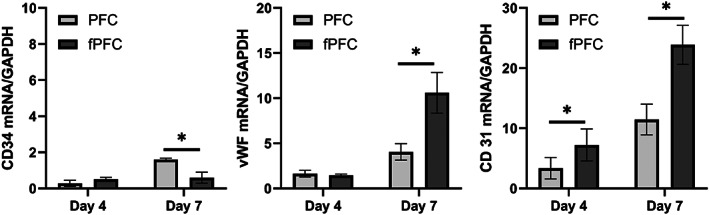
Gene expression of ECFCs on PFC and fPFC electrospun nanofiber coated coverslips at day 4 and day 7. Results for cells on fPFC and PFC were normalized to gene expression levels on tissue culture plastic (TCP). *p < .05.

From Figure 8, it can be seen that both vWF and CD31 were upregulated with increased culture time. CD34 expressed by ECFCs was reduced by 50% for both PFC and fPFC. At day 4, vWF expression increased twofold in cells cultured on PFC and fPFC compared with cells cultured on TCP. CD31 was upregulated about threefold in cells cultured on PFC and about sevenfold on fPFC compared with cells cultured on TCP. At day 7, the expression level of CD31 and vWF was significantly higher than on day 4. ECFCs cultured on fPFC had a 11‐fold increase in expression of vWF while cells on PFC had about a fourfold increase compared with cells cultured on TCP. Cells cultured on fPFC had significantly greater expression of CD31 compared with cells cultured on PFC. In general, based on the genetic markers measured, the results demonstrate enhanced differentiation of ECFCs toward an endothelial cell lineage on both PFC and fPFC and a significantly higher effect as a result of the presence of syndecan‐4 and SDF‐1α.

**FIGURE 9:**
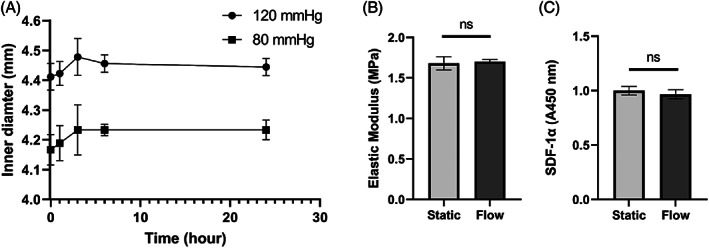
fPFC vascular graft evaluated in a custom‐built pulsatile flow bioreactor (see Video S1). (A) Inner diameter change over 24 h measured with ultrasound in the pulsatile flow bioreactor at 80 and 120 mmHg (see Video S2), (B) measurements of elastic modulus, and (C) ELISA quantification of SDF‐1α.

To determine if the SDF‐1 bound on PFC was resilient to vascular flow and pressure, conduits of the appropriate size of vessels that typically used in CABG surgeries were fabricated using fPFC. These conduits were tested in a custom‐built bioreactor.^32^ The initial ID of fPFC conduit was 4 mm. After being conditioned in the pulsatile flow bioreactor for 24 h, the ID did not change with alternating 80 and 120 mmHg pressure as shown in Figure 9A. With over 80,000 relaxation and stress cycles, no deformation, or signs of weakened mechanical properties such as wall bulging or weakening were observed. Elastic modulus of fPFC was 1.68 ± 0.08 MPa and 1.70 ± 0.03 MPa before and after being conditioned for 24 h (Figure 9B). Dynamic radial compliance of commercially available Dacron and PTFE grafts has been reported to be 1.9 ± 0.3 and 1.6 ± 0.2 (%/100 mmHg).^37^ fPFC had a compliance of 13.7 ± 0.4 (%/100 mmHg), which was closer to internal mammary artery (11.5 ± 3.9 (%/100 mmHg)^38^) than commercial products.

At the end of the 24‐h pulsatile pressure treatment, the ionically bound SDF‐1α remained on the material after being conditioned under physiological flow. No statistical difference between the amount of SDF‐1α remained on fPFC under flow and static conditions as shown in Figure 9C.

## 4 DISCUSSION

Engineered scaffolds designed to repair and regenerate a variety of tissues have become standard in many wound care protocols. Functionalization of scaffolds with biochemical and biophysical cues is critical to create a microenvironment to attract stem cells, a key event for tissue repair and regeneration. This study was designed to evaluate if functionalization of an endovascular biomaterial PFC, with syndecan‐4 and SDF‐1α would facilitate the adhesion, spreading and differentiation of ECFCs, processes critical in the formation of an intact endothelium for vascular repair and regeneration. The findings of this study suggest that functionalization with syndecan‐4 in combination with SDF‐1α significantly improves the recruitment of progenitor cells and supports their proliferation and differentiation.

Previous studies have shown that PFC is compositionally and mechanically similar to native vascular tissue and supports endothelial cell growth in culture.^22^ We have also found in a previous study that syndecan‐4 and SDF‐1α functionalized PFC (fPFC) significantly facilitated the binding of ECFCs compared with syndecan‐4 fPFC, SDF‐1α fPFC or non‐fPFC.^25^ In the current study, we confirmed and extended these findings to demonstrate more extensive cell spreading and differentiation of ECFCs if PFC was functionalized with syndecan‐4 and SDF‐1α. While only SDF‐1α was the exogenous growth factor used, the presence of syndecan‐4 on PFC may facilitate growth, spreading, and differentiation of ECFCs in multiple and versatile ways (see Figure 1). The molecular domain and structure of sydecan‐4 is similar to that found in endothelial glycocalyx, which coordinates the interaction with numerous cellular growth and signaling molecules and maintains hemostatic balance.^39^ As a heparan sulfate containing proteoglycan, syndecan‐4 has structural diversity to bind various molecules. Heparan sulfate oligosaccharides with specific binding sequences for over 400 molecules have been identified, and include a variety of growth factors, chemokines, cytokines, and anticoagulation molecules.^20^, ^21^, ^40^

The presence of syndecan‐4 on fPFC may function as a reservoir to capture and present a variety of cell‐secreted signaling and growth promoting molecules to surrounding cells, which will positively influence cell–cell and cell–extracellular matrix interaction. This is important for creating a local microenvironment conducive to endogenous regeneration instead of local delivery of stem cells or cell‐loaded scaffolds. These procedures typically suffer from drawbacks associated with high cost, prolonged and laborious ex vivo culture process, potential recipient rejection or permanent use of immunosuppressive therapies.^41^, ^42^

In the present study, ECFCs on fPFC were morphologically more spread than on PFC. For fPFC, the mean number of seeded cells per square centimeter was approximately 5 × 10^3^, which is comparable to the number of cells per square centimeter reported on vein graft surface (3.1 × 10^3^).^43^ Previous clinical studies have shown patients with low circulating ECFC counts have increased risks of vascular damage and endothelial dysfunction, and usually suffer from cardiac events such as atherosclerosis.^44^ These findings suggested insufficient numbers of cells associated with implanted cell‐free grafts may be a significant risk factor for atherosclerosis. Larger cell spreading area may enable the scaffold to be covered with fewer cells in CABG surgery, which is especially important for patients with low numbers of circulating ECFCs. In the present study, both vWF and CD31 expression was found to be significantly higher in cells cultured on fPFC than PFC. These results demonstrate the improved accelerated endothelial differentiation of ECFC on functionalized scaffold material. Enhanced ECFC differentiation may accelerate the maturation of endothelial cells, which is critical for vascular repair and the formation of a functional endothelium.^45^

AMD3100, a CXCR4 antagonist commonly used for blocking CXCR4 was applied to study if the interaction of SDF‐1α with CXCR4 on ECFCs alone contributed to increased number of captured cells on fPFC. Only a small effect was observed under dynamic binding condition, and increased dosage of AMD3100 showed comparable results. Similar results have been previously reported.^31^ A potential explanation may be the presence of multiple binding sites for cell growth and signaling molecules on syndecan‐4, enabling more complex interactions of fPFC with ECFCs.^31^ Potential mechanisms resulting in increased cell attachment may include the interaction of cell surface receptors and cell‐secreted growth‐promoting and signaling molecules with syndecan‐4 in additional to the binding of SDF‐1α to CXCR4 on ECFCs.

Selective binding of ECFCs by fPFC was evaluated by mixing labeled ECFCs with a mixed population of peripheral blood leukocytes at different ratios. In the present study, ECFCs bound to fPFC selectively in the presence of competing leukocytes and a small influence on binding was observed in the presence of a large excessive number of leukocytes. These studies were under static instead of dynamic blood flow and thus may overestimate any influence in vivo by circulating leucocytes. In addition, both the number of CXCR4 receptors and the binding affinity of the receptors have been reported to be greater on ECFCs compared with the other competing cells present in the blood stream.^46^ This may favor the binding affinity of ECFCs to fPFC under physiological blood flow conditions.

The fluid WSS sensing by the endothelial layer in human coronary arteries has been reported to range from 3.3 to 12.4 dynes/cm^2^.^47^, ^48^ For patients with CADs, the average WSS over the plaques has been reported to be 42% higher than the healthy region.^49^ For CABG surgery, WSS is greater at the anastomotic site and is relatively low along the bed of the graft (up to 11 dynes/cm^2^).^50^ To assess whether fPFC could capture ECFCs under physiological relevant shear stress conditions, the binding of ECFCs was evaluated at 1 and 10 dynes/cm^2^. These results demonstrated significantly reduced number of adherent ECFCs at higher versus lower shear stress. However, fPFC consistently captured significantly more cells than PFC at both low and high shear stress conditions. It is expected that during the in vivo vascular remodeling process, fPFC would facilitate the binding of ECFCs and accelerate the time to regenerate an intact endothelial lining.

Based upon clinical translational studies in large animal models, the normal amount of time for endothelialization on acellular vascular graft is approximately 4 weeks.^51^, ^52^, ^53^ Using the cell captured rate derived from present study, it is estimated that the time required for a small diameter fPFC vascular conduit to be sufficiently endothelialized would be approximately 2 weeks. A preclinical study of small intestine submucosa grafts coated with heparin‐bound VEGF in an ovine model demonstrated the adhesion and spreading of cells on lumen after 1 week of implantation, which developed into a confluent monolayer within 1 month.^50^ Using fPFC, more ECFCs may be captured from the blood stream potentially reducing the time required for endothelialization.

In addition to the essential role of circulating ECFCs, other cell sources for endothelialization have also been identified. Circulating monocytes have been shown to differentiate into endothelial cells and contributed to the formation of an intact endothelium in vivo.^35^ Mature endothelial cells also contribute to the endothelial lining on acellular scaffold material. Known as transanastomotic ingrowth, the host intima grows from anastomosis sites toward the implanted graft material as a response to the injury generated.^54^ Biomaterial surfaces such as those of fPFC favoring cell–material interaction may promote vascular repair and regeneration processes by facilitating adhesion, proliferation, and differentiation of a variety of other cell types associated with endothelialization.

Engineering biomaterials that will function and adapt to high arterial pressure and pulsatile flow are among the major challenges in vascular tissue engineering.^55^ In order to simulate conditions under which fPFC might be expected to perform upon eventual clinical use, tubular conduits were fabricated and tested. It is well known that saphenous vein grafts used in CABG surgeries dilate after being exposed to high arterial pressure. The fPFC conduits remained intact with no dilations or aneurysmal formations after being exposed to high pressure and repeated cycles of stress and relaxation in an ex vivo pulsatile flow bioreactor. In addition, the dynamic compliance of fPFC indicated it was similar to native internal mammary arteries. As opposed to commercialized Dacron and PTFE grafts having compliance mismatches with native vascular tissue. These results suggested that fPFC conduits were mechanically durable and able to withstand vascular pulsatile pressures following clinical use.

Native vascular extracellular matrix is formed from a complex organization of fibrous proteins and proteoglycans.^54^ The highly porous and interconnected microfibrillar nanostructure not only provides spatial residency and structural support for endogenous cells, but also serves as a reservoir to retain environmental cues that are essential in guiding cellular recruitment, proliferation, and differentiation. Many acellular vascular biomaterial scaffolds are fabricated using electrospinning fabrication protocols. The electrospun nanofiber 3D structures and alignments are very similar to the nanoscale dimension of native vascular extracellular matrix, which make electrospun biomaterials mechanically durable and resistant to vascular flow and pressure.^56^ In addition, the stability of the functionalized layer provides the strength of binding and thus the ability to withstand physiologically environment. The results of the current study have shown that ionically bound SDF‐1α remains on the material and is resilient to vascular pressure and flow. This finding was in agreement with the high binding affinity of SDF‐1α to heparan sulfate (*K*_*d*_ = 30 nM) reported in literature.^57^ While this study used ECFCs to evaluate the functionalized biomaterial for endovascular repair, these findings can be translated to other scaffolds used thus relate to regenerate a variety of tissues.

## 5 CONCLUSION

In this study, syndecan‐4 and SDF‐1α functionalized (fPFC) enhanced the binding, spreading and differentiation of ECFCs, all functions important in the formation of a nonthrombogenic endothelium following intravascular use. fPFC was stable and functional under high and low levels of vascular shear stress. fPFC fabricated as small diameter conduits and tested in a bioreactor were stable over time under physiological conditions of pulsatile blood flow. Syndecan‐4 functionalized biomaterials may provide a novel solution to promote tissue repair and regeneration process. The molecular diversity of oligosaccharide sequences on syndecan‐4 may provide flexibility to design acellular scaffold biomaterials to repair a variety of tissues.

## Supporting information


Video S1.

Video S2.

## Data Availability

The data that supports the findings of this study are available in the supplementary material of this article.
